# Association between triglyceride glucose index and H-type hypertension in postmenopausal women

**DOI:** 10.3389/fcvm.2023.1224296

**Published:** 2023-11-01

**Authors:** Zihao Ding, Shihong Du, Yi Yang, Tong Yu, Xiuqin Hong

**Affiliations:** ^1^ Clinical Epidemiology Research Office, The First Affiliated Hospital of Hunan Normal University, Changsha, China; ^2^ Key Laboratory of Molecular Epidemiology, Hunan Normal University, Changsha, China; ^3^ Department of Epidemiology and Statistics, College of Medicine, Hunan Normal University, Changsha, China; ^4^ Research Department, The First Affiliated Hospital of Hunan Norma University, Changsha, China

**Keywords:** H-type hypertension, triglyceride glucose index, insulin resistance, postmenopausal women, cross-sectional study

## Abstract

**Background:**

Recent studies have reported better predictive performance of triglyceride glucose (TyG) index for cardiovascular events in women, however, whether this association persists in postmenopausal women is inconclusive. We investigated the association between TyG index and H-type hypertension (HHT) in postmenopausal women.

**Methods:**

1,301 eligible women with hypertension were included in this cross-sectional study. Concomitant homocysteine levels >10 μmol/L were defined as H-type hypertension. The TyG index was calculated as ln [triglycerides (mg/dl) × fasting glucose (mg/dl)/2]. Multivariable logistic regression models and restricted cubic spline models were used to assess the association between TyG index and H-type hypertension in postmenopausal women, and subgroup analyses were performed for potential confounders.

**Results:**

Of the 1,301 hypertensive patients, 634 (48.7%) participants had H-type hypertension. In each adjusted model, TyG index was significantly associated with the risk of H-type hypertension. each 1-unit increase in TyG index was associated with an increased risk of H-type hypertension in all participants (OR = 1.6; 95% CI, 1.3–2.0; *P* < 0.001), and there was a linear relationship between TyG index and H-type hypertension (*P* for linear trend < 0.001).

**Conclusion:**

TyG index is positively associated with H-type hypertension in postmenopausal women, suggesting that TyG index may be a promising marker for H-type hypertension. By controlling lipid levels and blood glucose levels, it may help prevent H-type hypertension in postmenopausal women.

## 1. Introduction

As the most common cardiovascular disease, hypertension affects more than 1.278 billion people worldwide aged 30 to 79 years (1). Hypertension with homocysteine (Hcy) levels above 10 μ/L suggests H-type hypertension (2). In China, more than 70% of hypertensive patients have elevated plasma homocysteine levels (3). A cross-sectional study showed that hyperhomocysteinemia(HHcy) and hypertension are two independent, modifiable risk factors that add up to an increased risk of cardiovascular and cerebrovascular events (4). Therefore, early intervention of H-type hypertension is quite important.

Insulin resistance (IR) is a well-known predictor of many cardiovascular diseases (CVD) (5, 6). A hyperinsulin-normoglycemic clamp is the gold standard for measuring IR, but it is too expensive and complex to be used in clinical settings. Recently, triglyceride glucose (TyG) index, calculated from triglycerides (TG) and fasting glucose (FPG), has been proposed as a reliable alternative marker for IR (7). The TyG index is more sensitive and specific for the diagnosis of IR than past alternatives such as the homeostatic model insulin resistance index (HOMA-IR) and TG/HDL-C (8). The association between TyG index and hypertension has been demonstrated in relevant epidemiologic studies (9, 10).

As estrogen levels decline in postmenopausal women, cardiovascular diseases such as hypertension and hyperhomocysteinemia are more prevalent (11). H-type hypertension is more prevalent in postmenopausal women than in premenopausal women and men of the same age (12, 13). In addition, in postmenopausal women, IR is more likely to result from weight gain or abdominal obesity (14). According to previous studies, TyG index has better predictive efficacy in women than in men when it comes to major adverse cardiovascular events (15). This may be due to increased insulin resistance and decreased estrogen levels in postmenopausal women, but validation is still lacking. Thus, this cross-sectional study explores the relationship between TyG index and H-type hypertension among postmenopausal women.

## 2. Materials and methods

### 2.1. Study population

This cross-sectional study included 1,301 female patients treated at Hunan Provincial People's Hospital between January 2021 and January 2023 without missing data. Participant inclusion criteria: (1) postmenopausal women (cessation of menstruation for more than 1 year); (2) Diagnosis of essential hypertension; (3) cooperation with the survey and signing of informed consent form. Exclusion criteria: (1) participants who had used medications that could impact their blood pressure and homocysteine levels; (2) Patients who are unable to accurately describe their own state, such as mental illness, speech problems, etc.

All study participants signed an informed consent form before taking part in the study, which was evaluated and approved by the Medical Ethics Committee of Hunan Normal University (No. 034/2017). After data collection, all participant information is kept anonymous and each participant is given a code. No names are ever published in any way.

### 2.2. Sample size calculation

Sample size calculations were based on the prevalence of H-type hypertension among hypertensive patients using a single population proportion sample size formula. According to the literature review, the proportion of H-type hypertension in the Chinese hypertensive population was 73% (3), and a minimum sample size of 1,250 cases was required for the study, assuming an *α* of 0.05, a *β* of 0.80 and a 95% confidence interval.

### 2.3. Data collection and definitions

The data collection consisted of questionnaires, physical examination and laboratory tests. The questionnaire was administered by a uniformly trained investigator by face-to-face questioning, which included basic information, smoking history, drinking history, diet and physical activity.

Educational background was categorized into four groups: primary school and below, junior school, senior school, and college and above. Single or married were the two categories for marital status. Patients' level of regular activity was assessed based on how many times they worked out each week: 0 represented no exercise, 1–3 represented irregular exercise, and >3 represented regular exercise. We defined current alcohol intake as having at least two drinks per week and current smoking as continuously or cumulatively smoking one or more cigarettes per day for six months. The definition of covariates was informed by a previous questionnaire designed by the subject group for a cross-sectional survey of H-type hypertension (16).

Measurements of height, weight, waist circumference, and blood pressure are all part of a physical examination. A trained nurse used a conventional mercury sphygmomanometer to take three blood pressure readings at baseline, following a strategy that was developed from the American Heart Association's guidelines. A systolic blood pressure (SBP) ≥ 140 mmHg and/or a diastolic blood pressure (DBP) ≥ 90 mmHg were considered to be a diagnosis of hypertension (17). Patients with H-type hypertension were those who were diagnosed with essential hypertension and had a Hcy level >10 mol/L (18).

Within 24 h of admission, we took 5 ml of fasting venous blood and sent it to the Hunan Provincial People's Hospital's Department of Laboratory Medicine's fully automated biochemical analyzer to measure triglycerides (TG), fasting blood glucose (FPG), total cholesterol (TC), low-density lipoprotein cholesterol (LDL), high-density lipoprotein cholesterol (HDL), alanine transaminase (ALT), serum cr (Scr) and estimated glomerular filtration rate (eGFR).

### 2.4. Triglyceride-glucose index calculation

The values for glucose and triglycerides were converted from mmol/l to mg/dl. (multiplied by 18.020 and 88.545, respectively) (19). Triglyceride-glucose index was calculated as ln [triglycerides (mg/dl) × fasting glucose (mg/dl)/2] (20).

### 2.5. Statistical methods

Categorical variables were presented as numbers (*n*) and percentages (%), and the *χ*2 test was used to compare group differences. Data for normally distributed continuous variables are expressed as mean ± standard deviation (SD), and *t*-tests were used to compare differences between groups. Data for continuous variables with skewed distribution were expressed as median (interquartile spacing), which was examined by the Mann-Whitney test. Before applying the tests, its normality was checked with the Kolmogorov–Smirnov test, and its homoscedasticity was checked with Levene's test. We utilized multivariable logistic regression models to examine the association between Tyg quartile subgroups and risk of H-type hypertension after adjusting for known possible confounders between TyG and H-type hypertension as well as for covariates at *P* < 0.05 in univariate analysis. We created three models: model 1 was unadjusted; model 2 included adjustments for age, BMI, educational background, exercise, smoking and drinking history; model 3: The variables LDL, BUN, and eGFR were subsequently added to model 2. We observed no significant covariance between these variables (all VIF < 3) (Supplementary Table S1). To investigate the form of the association between the TyG index and H-type hypertension, we also used a restricted triple spline with three nodes (5th, 50th, and 90th percentiles), each truncated at the highest and lowest 0.5% of the TyG index. To determine whether relevant factors (age, body mass index, eGFR, smoking, alcohol consumption, and exercise history) influenced the association between TyG index and H-type hypertension, we performed subgroup analyses in which an interaction term was added to the logistic regression model. For different definitions of H-type hypertension (Hcy levels ≥ 15 μ/L), we further performed sensitivity analyses to explore whether the association continued to exist. All statistical analyses were performed using SPSS 25.0 and R 4.1.2, and the study was conducted using a two-sided test for *p*-values, with *p* < 0.05 considered significant.

## 3. Results

### 3.1. Baseline characteristics of included hypertension patients

In this investigation, we examined 1,301 postmenopausal women who had essential hypertension. The individuals' median TyG index concentration was 8.761 mg/dl (interquartile range, 8.291–9.167), and their median age (interquartile range) was 56.0 (51.0–66.0) years. By hypertension status, Table 1 displays the participant baseline characteristics. This investigation revealed 634 (48.7%) patients with H-type hypertension overall, with statistically significant variations in TyG quartile subgroups. In comparison to controls, obesity, low education, insufficient physical activity, and alcohol use were all linked to an elevated risk of H-type hypertension. Additionally, individuals with H-type hypertension had significantly lower eGFR and significantly higher levels of TG, LDL, Scr, BUN and FPG (*P* < 0.05).

**Table 1:** Baseline characteristics of included hypertension patients.

Characteristics	Total	Essential hypertension	H-type hypertension	*P*-value
TyG, *n* (%)	1,301	667	634	<0.001
<8.291 mg/dl	487 (37.4)	292 (43.8)	195 (30.8)	
8.291−8.761 mg/dl	227 (17.5)	122 (18.3)	105 (16.6)	
8.761−9.167 mg/dl	305 (23.4)	143 (21.4)	162 (25.5)	
≥9.167 mg/dl	282 (21.7)	110 (16.5)	172 (27.1)	
BMI, *n* (%)				<0.001
<18.5 kg/m^2^	50 (3.8)	30 (4.5)	20 (3.2)	
18.5–23.9 kg/m^2^	709 (54.5)	406 (60.9)	303 (47.8)	
≥24.0 kg/m^2^	542 (41.7)	231 (34.6)	311 (49.0)	
Education background, *n* (%)				<0.001
Primary school and below	23 (1.8)	16 (2.5)	7 (1.1)	
Junior school	268 (20.6)	156 (24.6)	112 (16.8)	
Senior school	440 (33.8)	195 (30.8)	245 (36.7)	
College and above	570 (43.8)	267 (42.1)	303 (45.4)	
Marital status, *n* (%)				0.098
Single	12 (0.9)	9 (1.4)	3 (0.5)	
Married	1,289 (99.1)	658 (98.6)	631 (99.5)	
Exercise, *n* (%)				<0.001
No exercise	512 (39.3)	212 (31.8)	300 (47.3)	
Irregular exercise	421 (32.4)	281 (42.1)	140 (22.1)	
Regular exercise	368 (28.3)	174 (26.1)	194 (30.6)	
Smoking history, *n* (%)				0.158
Never smokes	1,279 (98.3)	659 (98.8)	620 (97.8)	
Current or former smokers	22 (1.7)	8 (1.2)	14 (2.2)	
Drinking history, *n* (%)				<0.001
No	1,260 (96.9)	657 (98.5)	603 (95.1)	
Yes	41 (3.1)	10 (1.5)	31 (4.9)	
Age (years)	56.0 (51.0,66.0)	54.0 (49.0,58.0)	64.0 (55.0,72.0)	<0.001
WC (cm)	85.2 ± 24.0	84.3 ± 9.4	86.2 ± 32.9	0.152
TG (mmol/L)	1.4 (1.0,2.0)	1.0 (1.0,2.0)	1.0 (1.0,2.0)	<0.001
TC (mmol/L)	4.6 ± 1.2	4.6 ± 1.2	4.6 ± 1.1	0.249
LDL (mmol/L)	2.6 (2.1, 3.2)	2.6 (2.1, 3.1)	2.7 (2.1, 3.3)	0.006
HDL (mmol/L)	1.0 (1.0,1.1)	1.0 (1.0,1.2)	1.0 (1.0,1.0)	0.301
ALT (U/L)	16.0 (12.0,24.0)	16.0 (12.0,24.0)	16.0 (12.0,24.0)	0.635
Scr (μmol/L)	57.0 (50.0,24.0)	53.0 (47.0,60.0)	63.0 (54.0,77.0)	<0.001
BUN (mmol/L)	6.0 (4.6,8.1)	7.0 (4.4, 9.0)	6.0 (5.0,8.0)	<0.001
eGFR (ml/min)	98.0 (81.0,107.1)	104.0 (95.0,111.0)	86.4 (65.0,101.0)	<0.001
FPG (mmol/L)	5.0 (4.5,6.0)	5.0 (4.3,5.1)	5.0 (4.7,6.1)	<0.001

TyG index, triglyceride glucose index; WC, waist circumference; TG, triglyceride; TC, total cholesterol; HDL, high-density lipoprotein; LDL, low-density lipoprotein; ALT, alanine transaminase; Scr, serum creatinine; eGFR, glomerular filtration; BUN, blood urea nitrogen; FPG, fasting glucose.

### 3.2. Odds ratios of TyG index for H-type hypertension

Table 2 illustrates the connection between the TyG index and H-type hypertension. When compared to the lowest quartile of the TyG index, the ORs (95% CI) for H-type hypertension were 2.3 (1.7–3.1) in univariate analysis for the highest quartile. In a logistic regression model (model 3) adjusted for a number of confounders (age, BMI, educational background, exercise, smoking history, drinking history, LDL, BUN, eGFR), higher TyG index remained significantly associated with increased risk of H-type hypertension. The fully adjusted OR (95% CI) for the risk of HHT in quartile 4 vs. quartile 1 was 2.0 (1.4–2.9; *P* < 0.001). The TyG index was also examined as a continuous variable. According to model 3, postmenopausal women's chance of developing H-type hypertension increased by 0.6 (95% CI: 1.3–2.0; *P* < 0.001) per unit increase in TyG index. The association between TyG index and H-type hypertension remained significant even after adjustment for potential confounders.

**Table 2:** Association between TyG index and HHT in different models.

Tyg (%)	Model 1		Model 2		Model 3	
	OR (95% CI)	*P*-value	OR (95% CI)	*P*-value	OR (95% CI)	*P*-value
Per 1 unit increase (%)	1.7 (1.4–2.0)	<0.001	1.5 (1.3–1.9)	<0.001	1.6 (1.3–2.0)	<0.001
Quartile (%)						
*Q*1 (<8.291)	Reference		Reference		Reference	
*Q*2 (8.291–8.761)	1.3 (0.9–1.8)	0.118	1.1 (0.8–1.6)	0.818	1.0 (0.7–1.5)	0.948
*Q*3 (8.761–9.167)	1.7 (1.3–2.3)	<0.001	1.5 (1.2–2.1)	0.004	1.3 (1.0–2.1)	0.034
*Q*4 (≥9.167)	2.3 (1.7–3.1)	<0.001	1.7 (1.4–2.8)	0.001	2.0 (1.4–2.9)	<0.001
*P* for trend		<0.001		0.001		<0.001

Model 1: did not adjust.

Model 2: age, BMI, educational background, exercise, smoking history and drinking history.

Model 3: we adjusted for model 2 plus LDL, BUN, eGFR.

### 3.3. Linear relationship between TyG index and H-type hypertension

**Figure 1:**
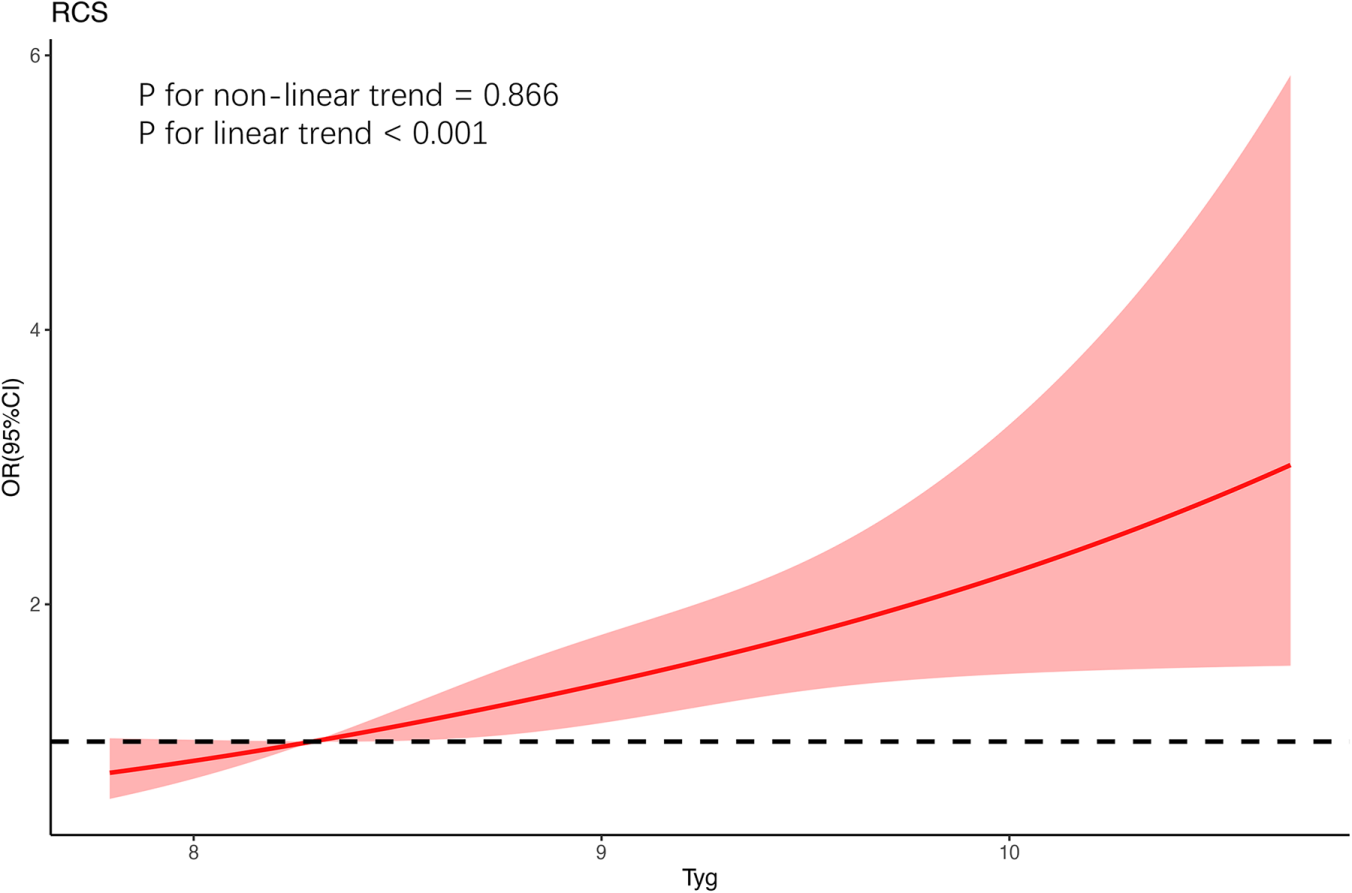
The restricted cubic spline for the association between postmenopausal women's TyG index and HHT. As a cut-off value for the first quartile, the reference value for TyG was established. For the model, restricted cubic spline regression was trimmed at the highest and lowest 0.5% of each TyG index, with the nodes in the three TyG indices at the 5th, 50th and 95th percentiles, and the variables for age, BMI, educational background, exercise, smoking and drinking history, LDL, BUN, and eGFR were all modified.

The TyG index and H-type hypertension were shown to be correlated linearly by restricted cubic spline regression models (*P* for linear trend < 0.001; Figure 1). The reference point is the cutoff value of the first quartile (8.291 mg/dl), and the nodes are located at the TyG index's horizontal range at the 5th, 50th, and 90th percentiles. The trend test (*P* < 0.001) between Tyg quartiles in the unadjusted model and the two adjusted models further illustrates the linear relationship, as shown in Table 2. The findings imply that a persistent rise in the TyG index increases the chance of postmenopausal women acquiring H-type hypertension.

### 3.4. Subgroup analysis

**Figure 2:**
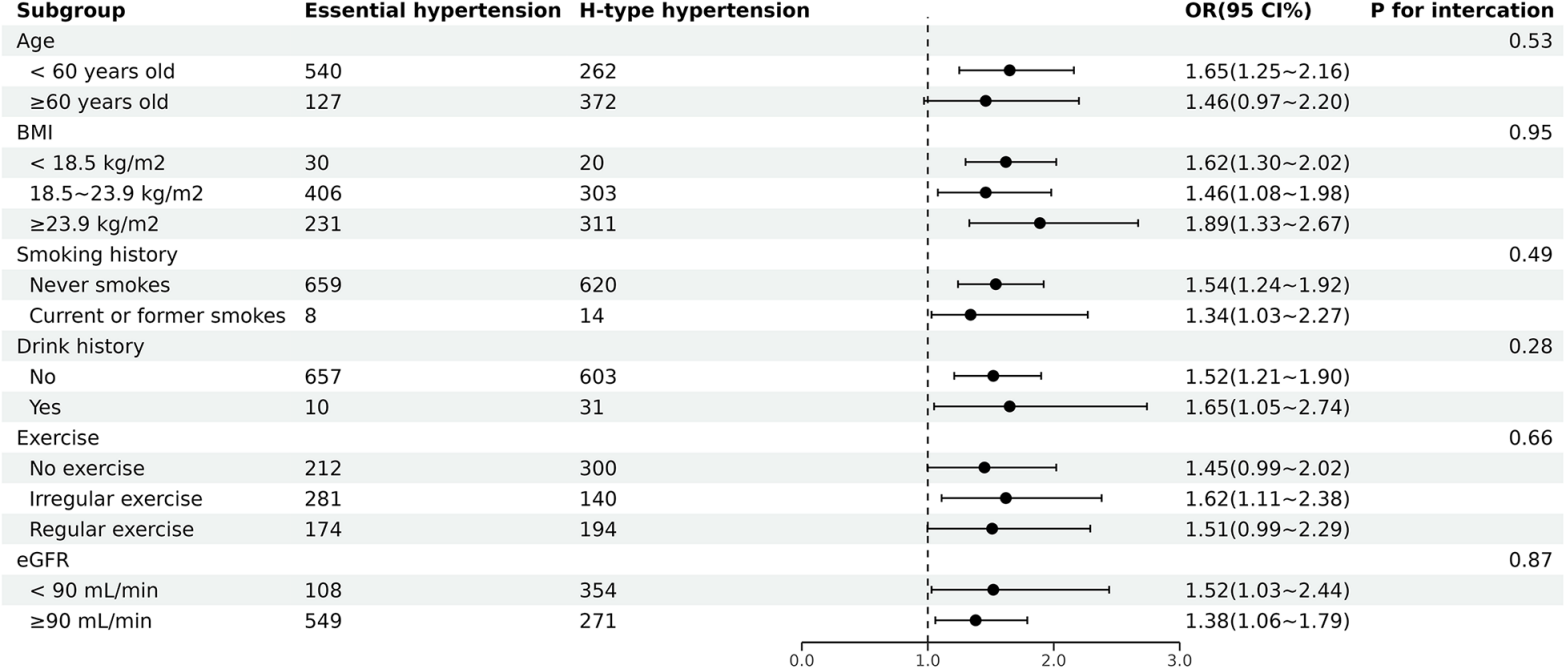
Subgroup analyses of the association between TyG index and H-type hypertension. ORs were adjusted for age, BMI, educational background, exercise, smoking history and drinking history, LDL, BUN, eGFR.

With regard to age, BMI, smoking history, drinking history, exercise, eGFR, subgroup analysis revealed that TyG index was positively associated with H-type hypertension. Futhermore there were no significant interactions between TyG index and these potential risk factors of interest for H-type hypertension (*P* > 0.05 for all interactions) (Figure 2).

### 3.5. Sensitivity analysis

As shown in Table 3, the results were stabilized (OR = 1.4; 95% CI, 1.1–1.9; *P* = 0.008) by sensitivity analyses for different definitions of H-type hypertension (Hcy levels ≥ 15μ/L).

**Table 3:** Sensitivity analysis of a hypertensive population with Hcy levels ≥15 μ/L.

Tyg (%)	Model 1		Model 2		Model 3	
	OR (95% CI)	*P*-value	OR (95% CI)	*P*-value	OR (95% CI)	*P*-value
Per 1 unit increase (%)	1.6 (1.3–1.9)	<0.001	1.4 (1.2–1.8)	0.001	1.4 (1.1–1.9)	0.008
Quartile (%)						
*Q*1 (<8.291)	Reference		Reference		Reference	
*Q*2 (8.291–8.761)	1.1 (0.7–1.6)	0.678	0.9 (0.6–1.4)	0.677	0.8 (0.5–1.4)	0.432
*Q*3 (8.761–9.167)	1.5 (1.1–2.2)	0.019	1.4 (0.9–2.1)	0.086	1.3 (0.8–2.1)	0.218
*Q*4 (≥9.167)	2.3 (1.6–3.2)	<0.001	1.9 (1.3–2.8)	0.001	1.8 (1.3–4.4)	0.012

Model 1: did not adjust.

Model 2: age, BMI, educational background, exercise, smoking history and drinking history.

Model 3: we adjusted for model 2 plus LDL, BUN, eGFR.

## 4. Discussion

In this cross-sectional study based on essential hypertension in postmenopausal women, we found that a higher TyG index was associated with the development of H-type hypertension in postmenopausal women. a positive dose-dependent association of TyG index with the risk of prevalence was obtained, after adjusting for possible confounders. Similar results were seen in subgroup analysis, highlighting the strength of these correlations even more.

There are two possible mechanisms by which excessive TyG leads to an increased risk of developing H-type hypertension. First, IR causes a decrease in the efficiency of glucose uptake and utilization by insulin in the body, resulting in compensatory secretion of excess insulin by the body. Excess insulin increases sympathetic nervous system activity and promotes the body to secrete more epinephrine and norepinephrine, which ultimately increases cardiac output and peripheral vascular resistance, and thickens vascular smooth muscle (20). In addition, IR has been shown to induce inappropriate activation of the renin-angiotensin-aldosterone system and the sympathetic nervous system (21), which may have a negative impact on renal function and, in turn, cause elevated Hcy levels. At the same time, elevated Hcy levels can cause increased secretion of various inflammatory factors, leading to abnormalities in the function of adipose tissue, promoting the production and secretion of resistin, which in turn promotes the occurrence of inflammatory reactions and insulin resistance (22, 23). Second, as two components of TyG, both TG and FPG are closely associated with the development of H-type hypertension. Hcy can increase oxidized LDL levels through oxidative modifications, prompting macrophages to take up large amounts of lipids to transform into foam cells and accelerate the deposition of cholesterol and TG on the vascular wall, while causing a large depletion of HDL, thus diminishing its ability to reverse cholesterol transport. This ultimately leads to the development of cardiovascular events, suggesting an association between HHcy and high TG (24). At the same time, a large number of epidemiological studies have shown that most hypertensive patients have dyslipidemia (25) and it has been demonstrated that lipid accumulation index (LAP) levels are higher in patients with H-type hypertension than in patients with non-H-type hypertension (26). Clinical observations have shown that hypertensive patients with elevated Hcy levels are positively associated with insulin resistance and diabetes mellitus (27–29), suggesting an association between FPG and H-type hypertension.

Previous studies have found gender differences in the relationship between TyG index and cardiovascular disease (15). As women age, ovarian function declines, estrogen decreases, and the body experiences a range of menopause-related symptoms (30). At the same time, as the protective effect of estrogen gradually decreases, the cardiovascular system of postmenopausal women undergoes significant changes, especially the risk of developing hypertension is significantly increased (31), and Hcy is also significantly higher in postmenopausal women (32). Epidemiological studies have demonstrated that menopause has become an independent risk factor for increased cardiovascular disease morbidity and mortality in women (33). Gender differences in the association between traditional cardiovascular risk factors can be explained by increased insulin resistance and lower estrogen levels in menopausal women, leading to an increased risk of cardiovascular disease (34). Our investigation reported similar findings suggesting that insulin resistance (high TyG index) is a risk factor for H-type hypertension in postmenopausal women. It is suggested that the development of H-type hypertension can be reduced by controlling FPG and TG and thus lowering the TyG index.

Despite these benefits and possible clinical applications, numerous study limitations must be taken into account when analyzing the findings. Firstly, in our cross-sectional survey, the possibility of residual confounding factors cannot be completely ruled out, this limits our ability to establish a causal relationship between TyG index and H-type hypertension in hypertensive patients. Therefore, to confirm the current findings, larger prospective investigations are required in the future. Finally, because we only examined postmenopausal women, it is not possible to extrapolate our findings to other populations.

## 5. Conclusions

In the present cross-sectional study, we found that the TyG index was independently associated with the development of H-type hypertension in postmenopausal women, and this simple index may be useful in identifying those at risk for H-type hypertension. We recommend that postmenopausal women control their lipid levels (TG) and blood glucose levels (FPG) levels, which may help prevent H-type hypertension.

## Data Availability

The original contributions presented in the study are included in the article/**Supplementary Material**, further inquiries can be directed to the corresponding author.
