# Supplementary material for: Association between triglyceride glucose index and H-type hypertension in postmenopausal women
Source: Front Cardiovasc Med. 2023 Nov 1;10:1224296. doi: 10.3389/fcvm.2023.1224296 (PMC10646504; doi:10.3389/fcvm.2023.1224296)
Supplement: Supplementary file 1 [file Datasheet1.docx]

**Supplementary table 1.** The collinearity diagnosis of fully adjusted models

| Characteristic | VIF |
| --- | --- |
| Age | 1.428 |
| BMI | 1.014 |
| Educational background | 1.074 |
| Exercise | 1.035 |
| Smoking history | 1.022 |
| Drinking history | 1.045 |
| LDL  BUN  eGFR | 1.055  1.113  1.278 |
| TyG index | 1.076 |

Data are presented as variance infation factor (VIF).
